# Ulcer of Lipschutz, a rare and unknown cause of genital ulceration

**Published:** 2018-03

**Authors:** A Moise, P Nervo, J Doyen, F Kridelka, J Maquet, G Vandenbossche

**Affiliations:** Hospital Notre Dame des Bruyères, CHU, Obstetrics and Gynecology, Rue de Gaillarmont 600, 4032 Chênée, Belgium

**Keywords:** genital ulceration, acute ulceration, young women, Lipchutz ulcer, diagnosis

## Abstract

Acute genital ulcers are painful and distressing to women. Lipchutz Ulcer is an uncommon disease that typically occurs in sexually inactive young women. The main differential diagnosis are sexually-transmitted or non-infectious diseases which cause genital or oro-genital ulcerations. This article aims to review the diagnosis of acute genital ulcers and, through a rare case of acute genital ulcerations, to discuss diagnostic procedures.

## Clinical history

A 13-year-old girl of Caucasian origin presented to the paediatric emergency room reporting acute vulvodynia, preceded by prodromal influenza-like symptoms (fever, tiredness, malaise) present for the past 48 hours.

The patient had no relevant medical history, nor recurrent buccal or genital apotheosis. She didn’t receive any regular medication. She was sexually inactive, and hadn’t applied any cream or anything else on the vulva. There was no trauma nor history of sexual abuse.

This is the first time she had experienced such symptoms.

**Figure 1:**
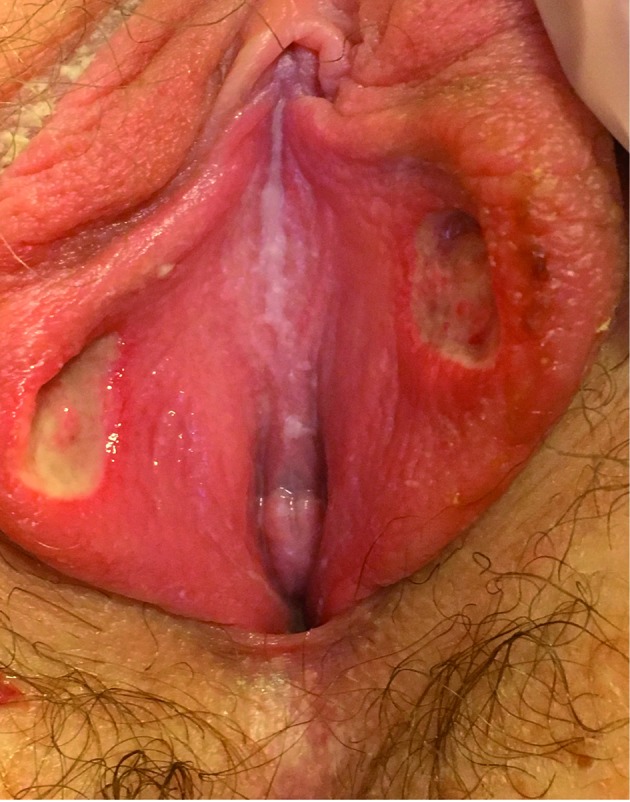
— Symmetric ulcerations of inner labia minor known as « kissing lesions », two deep, centimetric and fibrinous ulcerations.

The patient had an antalgic gait. Gynecologic examination showed her labia minor with two centimetric, fibrinous, soft ulcerations on their inner aspect. These lesions had a symmetrical appearance, known as « kissing lesions » (Figure 1). Furthermore, there was no abnormal leucorrhoea nor sign of trauma, but small adenopathies were palpated in the inguinal areas.

Intense pain caused stress for both the adolescent and her mother. Nevertheless, the patient didn’t present other clinical, extra-genital signs (no neurologic, cutaneous, ocular or gastrointestinal symptoms).

## Investigations

Blood count, viral (HIV, HBV, HCV, EBV, CMV), parasitic (toxoplasmosis) and treponemal (TPHA-VDRL) serologies as well as samples for further microbiological explorations (HSV PCR, anaerobic germs and mycosis cultures) have been performed. While results were pending, hospitalization in a paediatric ward was required with up to step II analgesic (paracetamol and tramadol) were administered in association with local hygiene (physiological serum) and wound care. An empirical antiviral treatment (acyclovir per os 200 mg 5X/ day) was initiated and the bladder was catheterized.

Performed explorations only showed an isolated inflammatory syndrome without hyperleukocytosis (CRP up to 20 mg/L). Viral and bacterial tests were negative and serologies didn’t bring any proof of recent infection. In light of these results, acyclovir was terminated. Ulcers eventually fully healed in a few weeks.

## Discussion

Acute vulvar ulceration, also referred to as a Lipschutz Ulcer, is a clinical diagnosis based on both:

— detailed history with acute painful genital ulcerations associated to fever in a sexually inactive, adolescent girl (Lipschütz, 1913)

— physical examination showing centimetric, fibrinous, soft necrotic ulcerations with red-violaceous border and inguinal adenopathies. Pathogenesis is unclear but a hypersensitivity reaction secondary to viral or bacterial infection with deposition of immune complex in the dermal vessels causing micro-thrombosis, and eventually leading to deep, necrotizing, painful, aphtoid lesions is suspected.

One hypothesis suggests a manifestation of EBV primary infection (Lorenzo en Robertson 2005).

The disease typically begins with prodromal influenza or mononucleosis-like symptoms followed by the onset of 1mm to 2,5cm, single or multiple (in this case, they are symmetrical, described as « kissing lesions »), vulvar ulcerations (Huppert, 2010).

The ulcerations typically involve the labia minor but can extend to the labia major, perineum vestibule and lower vagina. There is no risk of transmission. Spontaneous healing is complete in two to six weeks (this feature being a retrospective diagnostic argument), usually without scarring.

The clinical diagnosis is one of exclusion. As a matter of fact, sexually transmitted infections (HSV, treponema pallidum), auto-immune diseases (Behcet, IBD like Crohn disease...), trauma and other causes of acute genital ulcerations need to be excluded. Biopsy is often not required.

**Figure 2:**
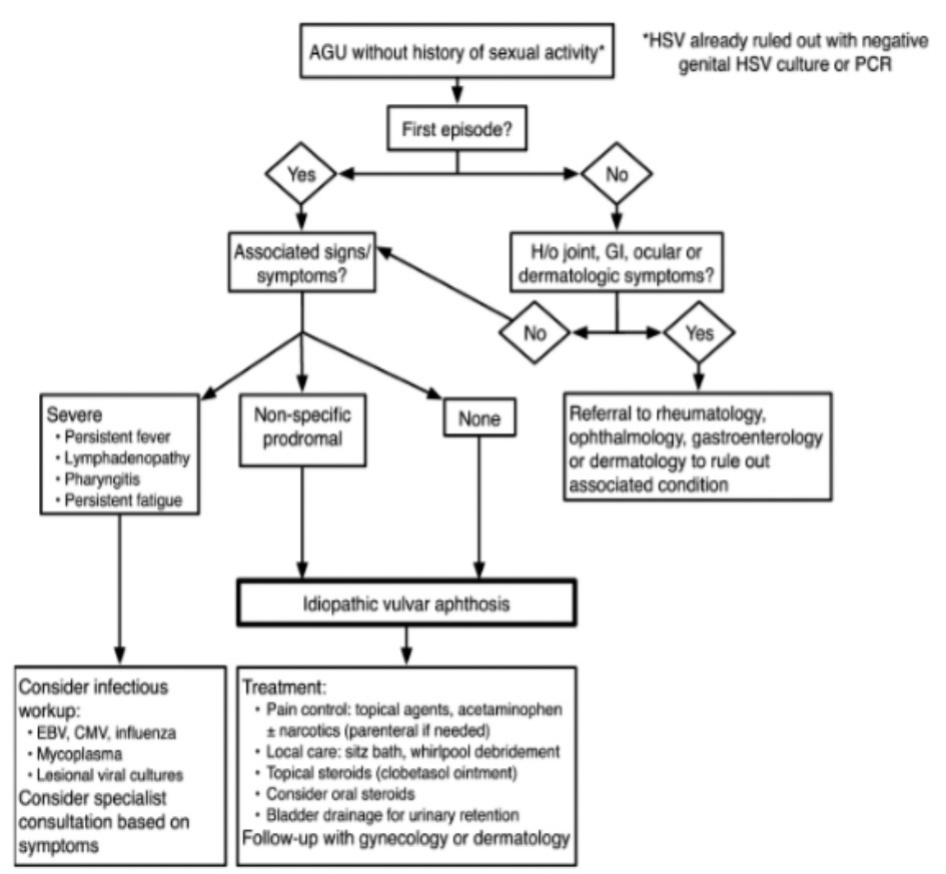
— Algorithm for evaluation and treatment of acute genital ulceration (AGU) in young women (Rosman et al., 2012) (HSV = Herpes Simplex Virus, PCR = Polymerase chain reaction, GI - gastrointestinal, EBV = Epstein-Barr Virus, CMV = Cytomegalovirus).

Rosman et al. have developed an algorithm for evaluation and treatment of acute genital ulcerations after HSV-infection exclusion (Figure 2) (Rosman et al., 2012).

Patient care consists of pain control including topical and systemic analgesia and wound care. Patient and parents should be reassured that the disease is benign and its lack of recurrence. In case of severe pain, hospitalization with bladder catheterization might be required to avoid any secondary urine retention (Bandow 2010).

The main differential diagnosis of Lipschutz ulcerations are sexually transmitted diseases and non-infectious diseases which cause genital or oro-genital ulcerations.

The most common diseases in this regard are (Robert, 2017):

- genital herpes
- HIV infection
- syphilis
- complex aphthosis
- Behcet’s syndrome
- Crohn’s disease
- pyoderma gangrenosum
- childhood vulval pemphigoid

## Conclusion

Lipchutz Ulcer is an uncommon disease that typically occurs in young, sexually inactive women.

The diagnosis is one of exclusion and other causes of genital ulcerations such as genital herpes, sexually transmissible infections (syphilis, HIV, ...) and auto-immune disease (Behcet’s syndrome, Crohn’s disease...) must first be ruled out.

Healing without scarring is spontaneous under proper analgesia and wound care, this feature being an important diagnostic argument.
